# Prompt PSA changes as a prognostic marker for response to PSMA-radioligand therapy

**DOI:** 10.1007/s00259-026-07772-y

**Published:** 2026-01-23

**Authors:** Wolfram A. Bosbach, Radi Saiyed Alsheikh, Nasir Gözlügöl, Federico Caobelli, Robert Seifert, Clemens Mingels, Mohamed Shelan, Dilara Akhoundova, Berna C. Özdemir, Jörg Beyer, Paul Cumming, Axel Rominger, Ali Afshar-Oromieh

**Affiliations:** 1 Department of Nuclear Medicine, Inselspital, Bern, University Hospital of Bern, Bern, Switzerland; 2 Department of Radio-Oncology, Inselspital, Bern, University Hospital of Bern, Bern, Switzerland; 3 Department of Medical Oncology, Inselspital, Bern, University Hospital of Bern, Bern, Switzerland; 4 School of Psychology and Counselling, Queensland University of Technology, Brisbane, Australia

**Keywords:** PSMA radioligand therapy, PSMA kinetics, Early treatment response, Metastatic castration-resistant prostate cancer, [177Lu]Lu-PSMA-617, Overall survival prediction

## Abstract

**Introduction:**

We investigated whether prompt changes in prostate-specific-antigen (PSA) levels within two days after the first cycle of prostate-specific-membrane-antigen radioligand therapy (PSMA-RLT) with [^177^Lu]Lu-PSMA-617 predicted treatment response and mean survival.

**Methods:**

In a retrospective study of 76 metastatic castration resistant prostate cancer (mCRPC) patients, we evaluated pretreatment PSA-values and their relative changes in PSA (dPSA) two days later. We tested for correlations between dPSA with long-term biochemical response (BCR) to treatment, using a priori criteria for relevant PSA decrease (dPSA < -10%), stable PSA (-10% ≤ dPSA ≤ + 10%) and relevant PSA increase (dPSA > 10%), along with evaluation of biochemical therapy outcome according to the Prostate-Cancer-Working-Group (PCWG3).

**Results:**

Two days after the first [177Lu]Lu-PSMA-617 cycle, 32 (42%) of the patientsshowed PSA decrease, of whom 19 (59%) had experienced a partial response according toPCWG3 criteria. Of the 37 patients with stable PSA, 17 (46%) showed partial response totreatment according to PCWG3 criteria. Among the seven patients with PSA increase, three(43%) showed partial response. Pearson correlation analysis showed statistically significantcorrelations between dPSA on day 2 and relative Nadir for the first two treatment cycles.Patients with PSA decrease or stable PSA compared to those with an increase of PSA two daysafter cycle 1 lived longer on average (399, 405 and 225 days, respectively).

**Conclusion:**

Compared to those with increased PSA levels, patients with decreased or stable PSA levels two days after the first [^177^Lu]Lu-PSMA-617 RLT cycle were more likely to have favorable biochemical response according to PCWG3 criteria and presented with a longer overall survival.

**Supplementary Information:**

The online version contains supplementary material available at https://doi.org/10.1007/s00259-026-07772-y.

## Introduction

Prostate cancer (PCa) is one of the most common cancers diagnosed worldwide among men, and is the eighth leading cause of cancer-related deaths [1], due to the generally poor treatment responses in metastatic disease. In the first- and second-line treatment of metastatic hormone-resistant prostate cancer (mCRPC), treatment consists of additional androgen pathway inhibitors (ARPI), chemotherapeutic agents, or palliative ${}^{223}$ Radium-dichloride treatment. For third-line treatment of mCRPC after treatment with ARPI and taxane chemotherapy, radioligand therapy (RLT) with the ^177^Lutetium-labelled ligand PSMA-617 (Pluvicto®) has recently received approval by the American Food and Drug Administration (FDA), the European Medicines Agency (EMA), and by Swissmedic [2].

Prostate specific membrane antigen (PSMA) is a class II membrane glycoprotein that is frequently overexpressed in the majority of prostate cancer cells [3], thus presenting a target for radioligand therapy of advanced PCa. There is specific indication for the application of PSMA-RLT in individuals with PSMA-positive mCRPC that has progressed despite ARPI and taxane chemotherapy.

A multitude of studies, including the phase III VISION-trial [4], have confirmed the efficacy of PSMA-RLT in the setting of mCRPC. Epithelial cells of the healthy prostate gland secrete the glycoprotein peptidase enzyme known as prostate specific antigen (PSA) into circulation. Although PSA levels between 4 and 10 ng/mL can be associated with benign conditions such as prostatitis, higher PSA levels are more likely to reflect PCa, although overlap exists and further diagnostic evaluation is required. Since its inception in the late 1980 s, test of serum PSA levels remains the cornerstone for PCa screening, serving as a guide for the decision to biopsy. Changes in serum PSA levels are indicative of treatment efficacy, disease recurrence, or progression [5, 6].

The preponderance of circulating PSA is normally bound to plasma proteins. In clinical trials and likewise in clinical routine, longitudinal PSA monitoring frequently serves to evaluate the response to PSMA-RLT [7, 8].

This approach calls for baseline PSA ($PS{A}_{bl}$) measurements for monitoring the individual therapeutic efficacy of PSMA-RLT. Clinicians generally categorize the PSA response in accordance with criteria recommended by the Prostate Cancer Clinical Trials Working Group 3 (PCWG3) [6].

While current PCWG3 recommendations suggest evaluation of PSA response no earlier than twelve weeks after the initiation of treatment [6], a more recent study of PSMA-RLT indicated that biochemical differentiation of therapy responders from non-responders might be possible as early as six weeks [9].

A response of declining PSA after 1–2 cycles of PSMA-RLT is a significant prognostic factor in mCRPC patients, predicting a further decline in PSA and a favorable clinical response [10, 11]; early responders showed improved overall survival rates compared to those with stable or increasing PSA levels after the first cycles.

This study recapitulated earlier findings that prompt PSA changes predict long-term biochemical response and patient survival [12, 13].

Since the clinical introduction of PSMA-RLT at our center in 2019, where brief hospitalization of such patients is a requirement according to our national regulations, we have adopted the determination of each patient´s $PS{A}_{bl}$ value on the day of radiopharmaceutical admission and upon their hospital discharge on the second day after each cycle. The aim of our study was to investigate whether prompt $\Delta \mathrm{\%}PSA$ levels within two days after radiopharmaceutical administration can serve as a very early predictor of treatment response.

## Material and methods

### Patients’ cohort

Our study retrospectively evaluated patients who had received at least one cycle of ^177^Lu-PSMA-617 RLT between August 2019 and January 2023 in their third-line treatment of mCRPC at the Department of Nuclear Medicine of the University Hospital of Bern, Switzerland. Further inclusion criteria were as follows: patients who have given written informed consent for the anonymized assessment of their medical data, in whom follow-up was possible for at least 1.5 years, who had PSA measurements on the day before the injection of [^177^Lu]Lu-PSMA-617 as well as two days thereafter, and patients who have received no other PSMA-based radiopharmaceutical than [^177^Lu]Lu-PSMA-617. ^177^Lu-PSMA-617 was provided by Novartis in a Managed Access Program for the majority of patients. The local ethics committee approved this retrospective study (project ID-2023–02053), and all subjects signed a written informed consent form.

### Study design

On the day of treatment (day 0) during each PSMA-RLT cycle, we obtained blood samples for evaluation of plasma PSA, assessment of hematologic and renal status, liver function tests, and alkaline phosphatase and blood biochemistry prior to ^177^Lu-PSMA-617 injection. Two days after each [^177^Lu]Lu-PSMA-617 (day 2), we re-evaluated the above-mentioned biomarkers as part of routine patient care before patient discharge. As part of our clinic’s follow-up standards, we requested that the patients undergo outpatient blood tests at two and four weeks after PSMA-RLT. Time interval between each PSMA-RLT cycle was 6–8 weeks, which was in accordance with the current EANM guidelines. During the longitudinal data collection, we systematically analyzed therapy continuity and dropouts, with recording of PSA levels across up to eight treatment cycles. Subsequently, those patients who had shown no obvious signs of progression according to PCWG3 criteria received further cycles of PSMA-RLT, while we switched the patients with progression to other treatment options.

### Treatment response as defined by PCWG3

Definition for the assessment of biochemical treatment response by PCWG3 is found in [6, 14, 15], see Fig. 2. Progression (PROG) was defined as a 25% increase in PSA from the nadir/baseline, or an absolute rise of 2 ng/mL, confirmed by a second measurement obtained at least 3 weeks later. Partial response (PR) was defined as a 50% decrease in PSA from baseline, also confirmed by a subsequent measurement after at least 3 weeks. In line with the original PCWG criteria [15], complete response (CR) was not reported; biochemical PSA response assessment therefore referred to PR. Early PSA rises occurring before 12 weeks were excluded from response evaluation (“early risers”). Stable disease (SD) was defined when neither PROG nor PR criteria were met.

### Statistical analysis

**Fig. 1:**
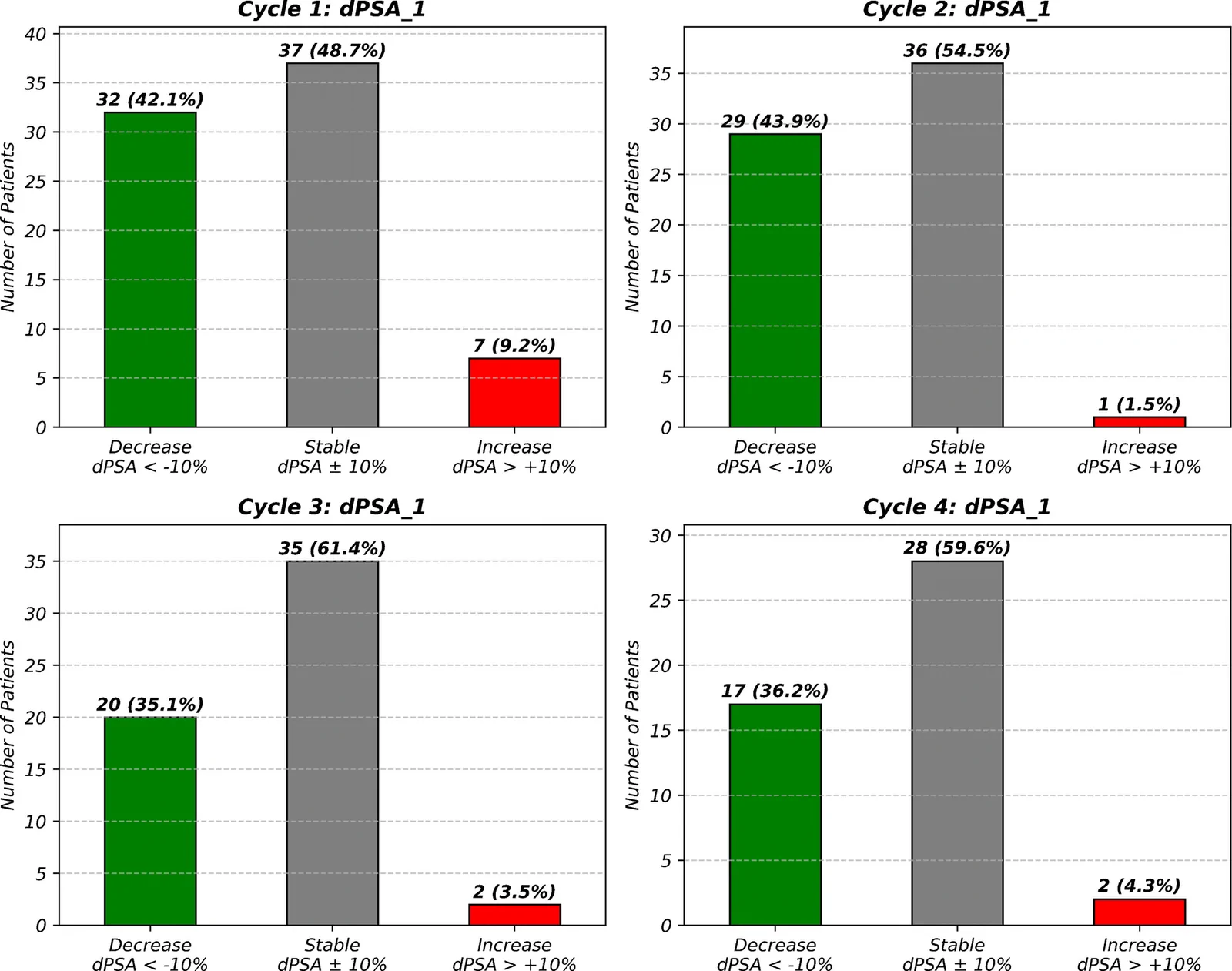
The bar plots depict the distribution of patients based on prompt changes in their prostate-specific antigen (PSA) levels, with categorization in into three groups: "decrease," "increase," and "stable" PSA levels, according to stringent thresholds of 10% changes relative to pre-cycle levels (Eq. 2). Each bar displays the number of patients, and their percentage representation within that cycle

**Fig. 2:**
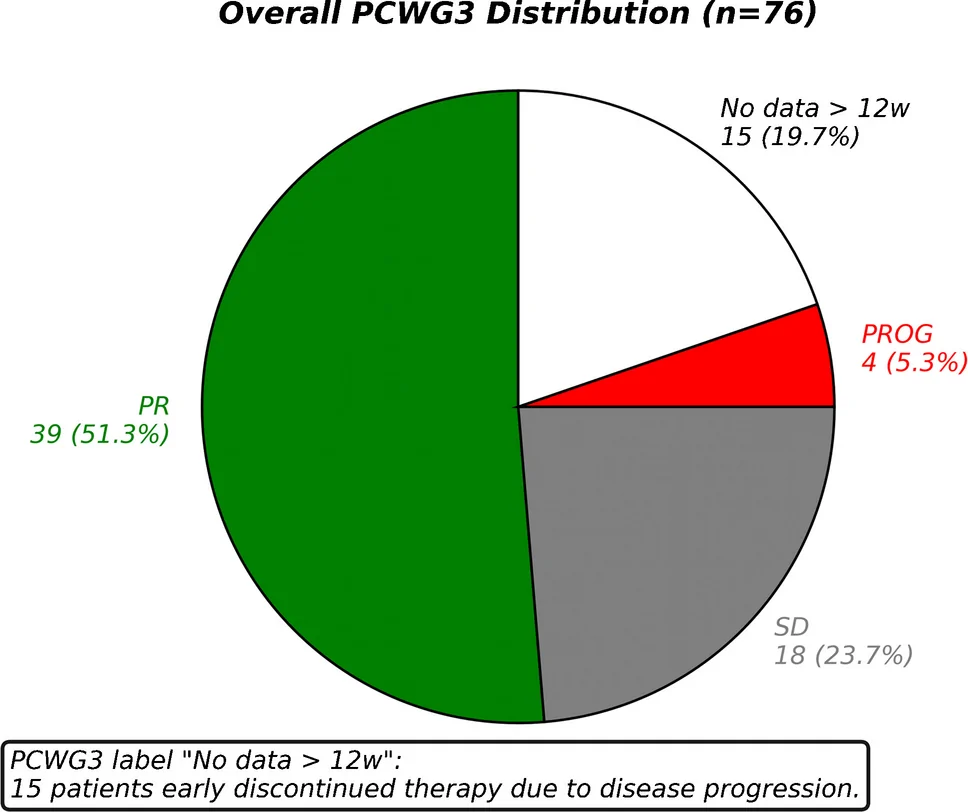
*O*verall PCWG3 status for all 76 patients, split into partial response (PR), stable disease (SD), progression (PROG), and without data past 12 weeks

All statistical analyses, figures, and tables were obtained through Python 3.11.7 (Anaconda, Inc.) code. Significance level alpha was set to 0.05 for the present study. From the four PSA values per cycle (therapy day, after 2 d, after 2w, after 4w), three dPSA values were calculated per cycle, Eq. 1. Those dPSA describe the relative change of PSA over the course of the respective cycle. dPSA were binned into three trend groups: increase (> 10%)/stable (± 10%)/decrease (< −10%), Eq. 2, see Fig. 1. The relative Nadir (rNadir in Eq. 3) was calculated as best biochemical response in comparison to the first PSA value of cycle 1 (Fig. 2).

1 $$\begin{array}{l}dPSA\_1=\;\frac{PSA\_2-PSA\_1}{PSA\_1}\\ \\ dPSA\_2= \frac{PSA\_3-PSA\_1}{PSA\_1}\\ \\ dPSA\_3= \frac{PSA\_4-PSA\_1}{PSA\_1}\\ \\dPSA\_1\;to\;dPSA\_3\;calculated\;per\;cycle\end{array}$$

2 $$dPSA \;\; trend:\;dPSA \left\{\begin{array}{l}>10\%:\;trend=\;increase\\\;\pm\;10\%:\;trend= stable\\\;<-10\%:\;trend=\;decrease\end{array}\right.$$

3 $$\begin{array}{l}rNadir=\;\frac{Nadir-PSA\_1}{PSA\_1}\\ PSA\_1\;of\;cycle\;1\end{array}$$

**Fig. 3:**
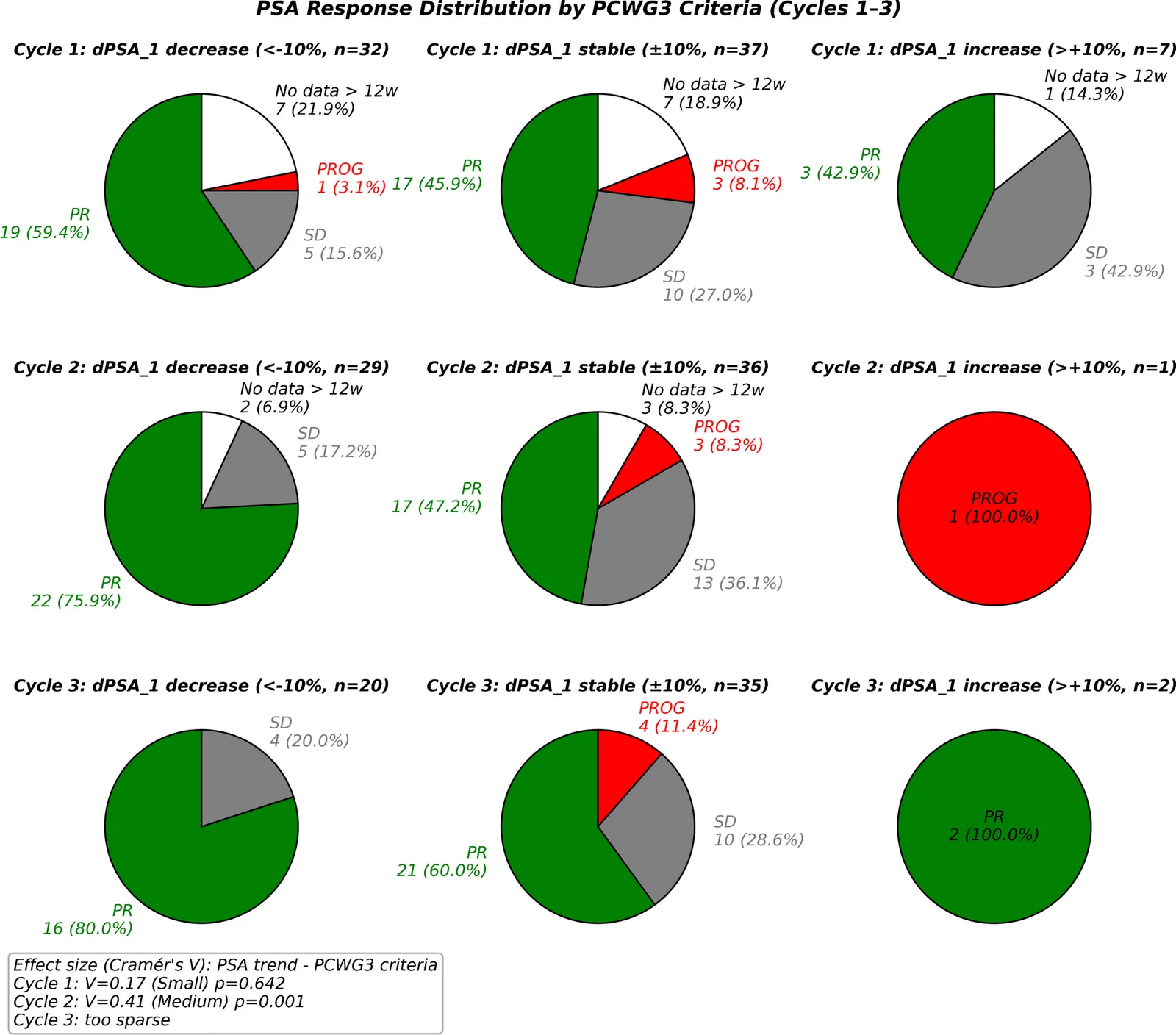
The pie charts depict the relationship between the PCWG3 therapy response and the relevant PSA changes across the first three treatment cycles, stratified by 10% thresholds for increased, unchanged, or decreased relative to the precycle baseline values (Eq. 2)

The association between the trend of dPSA_trend (Eq. 2) and PCWG3 response was evaluated using Cramér’s V as a measure of effect size (Fig. 3), [16, 17].

**Fig. 4:**
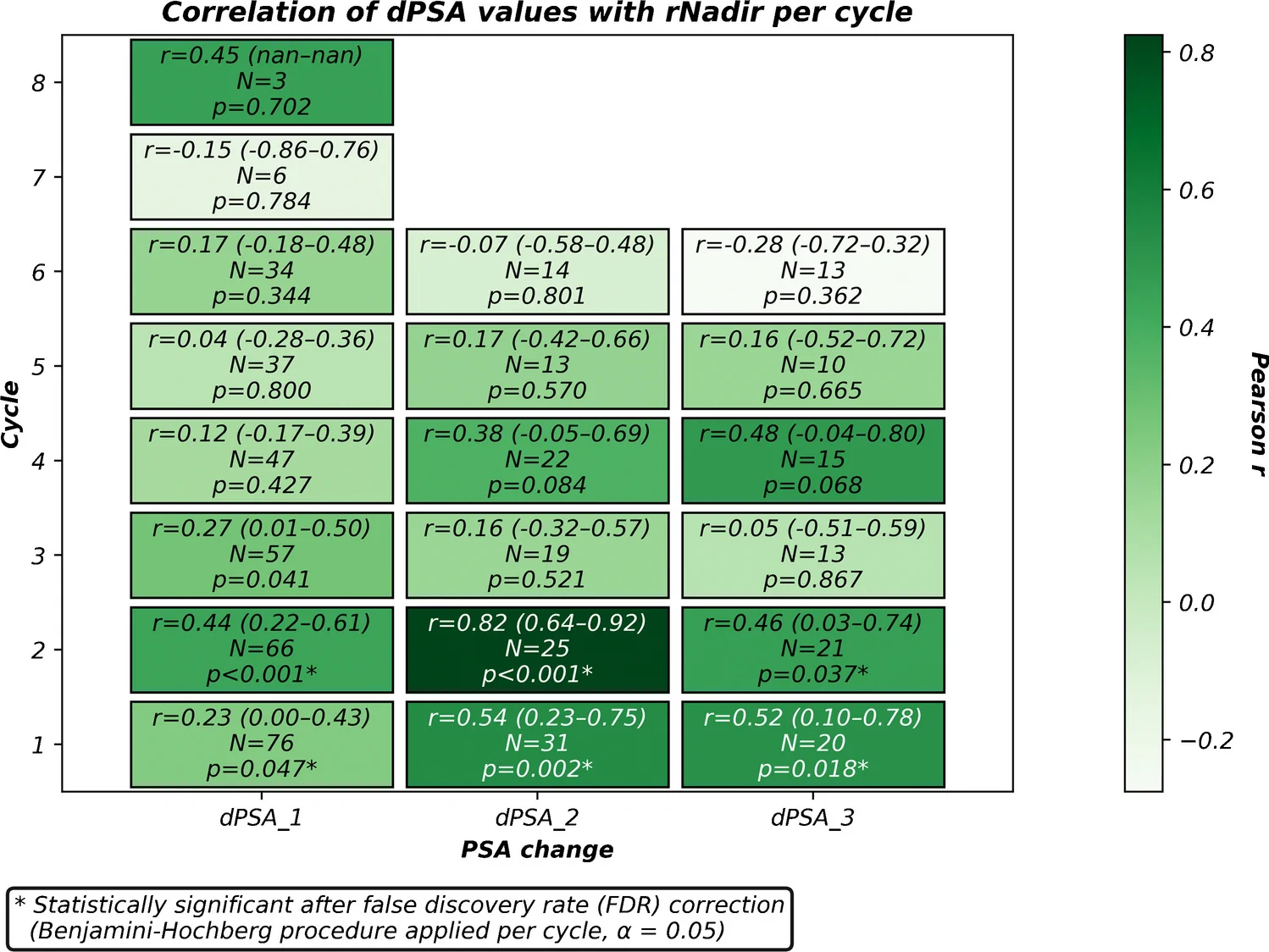
Correlation between relative PSA changes (dPSA_1 to dPSA_3) and relative PSA Nadir (rNadir) across treatment cycles. The Pearson correlation coefficients are given with 95%-CI, N, and p (p*: significance after false discovery rate (FDR) correction per cycle using the Benjamini–Hochberg procedure)

Pearson’s correlation coefficient (r) was calculated between dPSA (Eq. 1), and the rNadir (Eq. 3, Fig. 4, [18]). Only patients with non-missing paired values of these two variables were included in the correlation analysis. 95% CI for r were calculated using Fisher’s z-transformation (alpha = 0.05). To account for multiple significance testing, false discovery rate (FDR) correction using the Benjamini–Hochberg procedure was applied separately within each treatment cycle, with a significance threshold of alpha = 0.05 [19]. Statistically significant correlations after FDR correction were denoted by an asterisk (*) in Fig. 4.

**Fig. 5:**
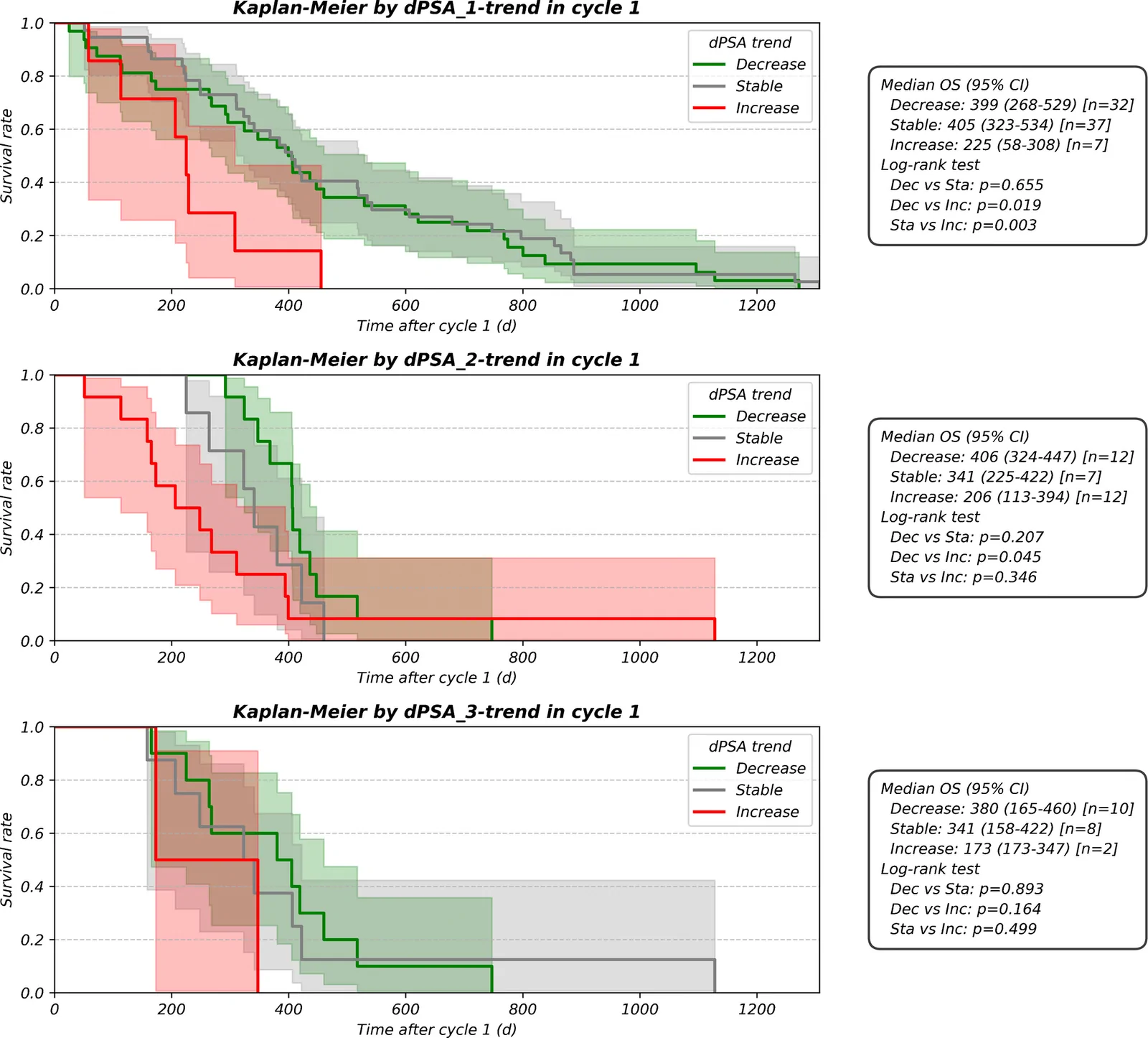
Kaplan–Meier survival curves illustrating the overall survival probabilities for groups of patients stratified by changes in PSA within the first treatment cycle. Group assignment derives from stringent thresholds for PSA response relative to baseline levels, thresholded for 10% changes: "decrease," "increase," and "stable" (Eq. 2). Survival probability is plotted over time (days), with shaded confidence intervals for each group

Overall survival (OS) was calculated for each patient as the time interval between the baseline PSA_1 of cycle 1 and the date of death. All patients were deceased at the time of analysis. Kaplan–Meier survival curves were generated to evaluate OS in relation to PSA kinetics during the first treatment cycle [20]. For each of the three dPSA of cycle 1 (Eq. 1), patients were categorized into one of three PSA trend groups (Eq. 3): Decrease, Stable, or Increase. Separate Kaplan–Meier plots were produced for each dPSA, each displaying survival curves for the three PSA trend groups (Fig. 5). Median OS, and corresponding 95% CI were extracted from the Kaplan–Meier estimates. Pairwise group comparisons were performed using the log-rank test to assess differences in survival distributions between trend categories.

**Fig. 6:**
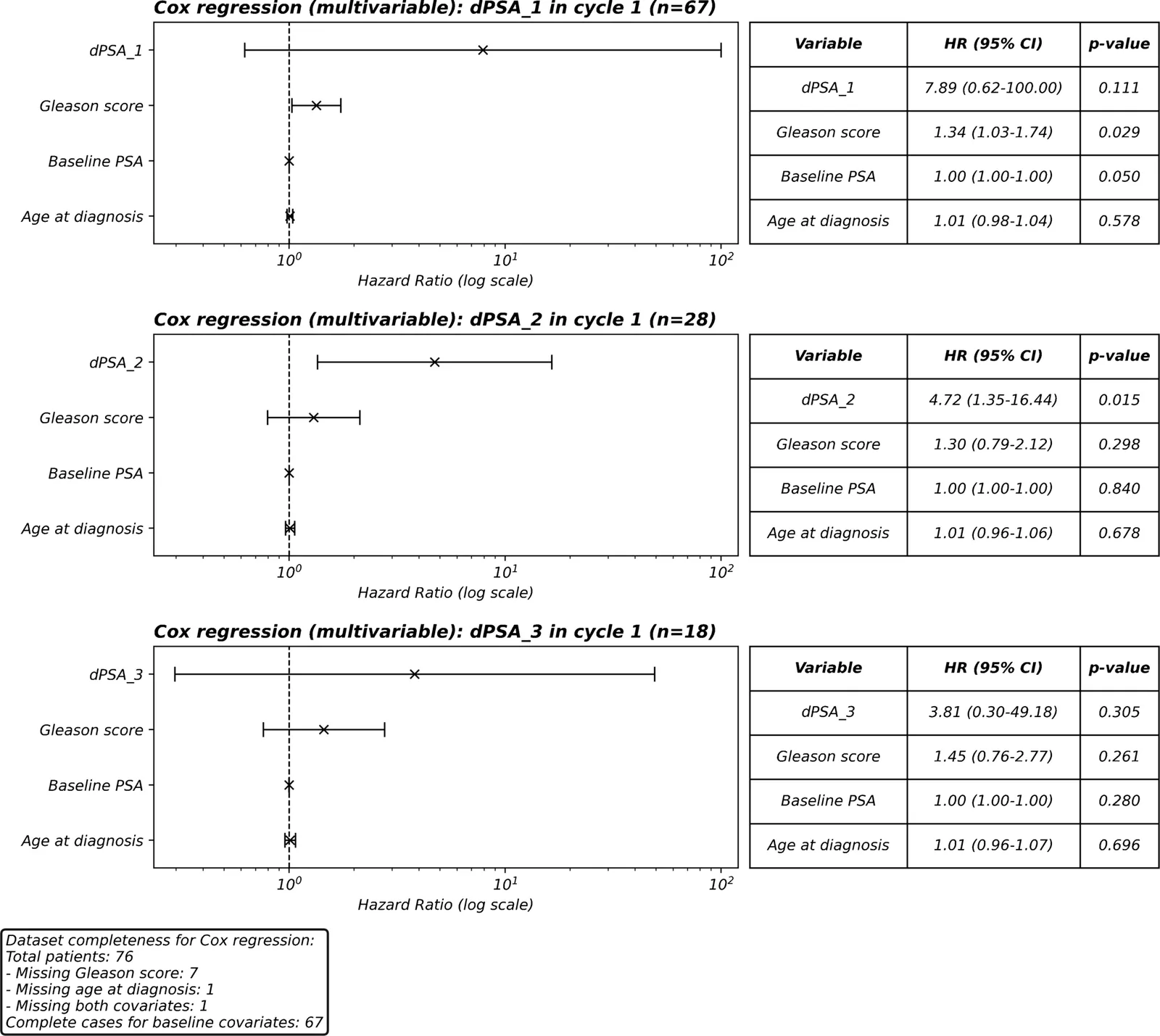
Forest plots of multivariable Cox proportional hazards models evaluating the association of early PSA changes (dPSA_1 to dPSA_3) with OS. Each subplot represents a separate model along with baseline covariates (Gleason score, baseline PSA, age at diagnosis). Hazard ratios (HR) and 95% confidence intervals (CI) are shown on a log scale for each covariate, with corresponding p-values displayed in the adjacent table. The vertical dashed line at HR = 1 indicates no effect

Multivariable Cox proportional hazards regression models were constructed [21]. Separate Cox models were fitted for each dPSA metric of cycle 1 (Eq. 1) as the main predictor variable. Each model was adjusted for established clinical covariates: Gleason score, baseline PSA, and age at diagnosis. Cases with missing values in any of the included covariates were excluded from the respective model. For each model, results were visualized as a forest plot showing Hazard ratio (HR, log scale) and 95% CI for all covariates within each model, Fig. 6.

## Results

A total of 76 patients were included in our analysis. They underwent in total 329 cycles of PSMA-RLT (mean = 4.3 cycles, median = 4 cycles). We present the detailed patient characteristics in Table 1.

**Table 1:** Patient Characteristics. This table summarizes the demographic, clinical, and treatment-related characteristics of the 76 metastatic castration-resistant prostate cancer (mCRPC) patients included in the study

Patients *n*		76
Age at cycle 1 [a]		mean 72, range 54–89
Gleason score n (%)	Gleason 6	2 (2.6%)
	Gleason 7	19 (25.0%)
	Gleason 8	15 (19.7%)
	Gleason 9	29 (38.2%)
	Gleason 10	3 (3.9%)
	Missing	8 (10.5%)
Number of cycles n (%)	1 cycle	10 (13.2%)
	2 cycles	10 (13.2%)
	3 cycles	9 (11.8%)
	4 cycles	10 (13.2%)
	5 cycles	4 (5.3%)
	6 cycles	26 (34.2%)
	7 cycles	3 (3.9%)
	8 cycles	2 (2.6%)
	9 cycles	1 (1.3%)
	11 cycles	1 (1.3%)

### PSA trend in cycle 1 to 4

On day 2 of the first cycle (dPSA_1), 32 patients (42.1%) were categorized as belonging to the decreasing group (Eq. 2). 37 (48.7%) were in the stable group, seven (9.2%) were in the increasing group. The corresponding numbers are shown in Fig. 1 for cycles 1 to 4. Suppl-Table 1 (all patients), and Suppl-Table 2 (discontinued patients) give the detailed lists of patients with dPSA categorized by trend.

### PCWG3 classification

In the total dataset of 76 patients, 15 (19.7%) discontinued therapy early before the end of week 12 due to disease progression. In the PCWG3 classification, they received no label, Fig. 2. From those patients who continued therapy beyond week 12, 39 (51.3%) showed PR, 18 (23.7%) SD, and 4 (5.3%) PROG.

The patient subgroups of Fig. 1 are shown split by PCWG3 status in Fig. 3. In Cycle 1 in the PSA decrease group, 19 (59.4%) achieved PCWG3 PR, while 5 (15.6%) had SD, and 1 (3.1%) experienced PROG. In contrast, the stable PSA subgroup exhibited PR in 17 (45.9%), SD in 10 (27.0%), and progression in 3 (8.1%) patients. By Cycle 2 within the subgroup of PSA decrease, 22 (75.9%) patients achieved PR, and 5 (17.2%) SD. The stable PSA group in cycle 2 showed 17 (47.2%) PR, 13 (36.1%) SD, and 3 (8.3%) PROG. Only one patient was assigned to the PSA increase group in cycle 2, who exhibited biochemical PROG (100%). At Cycle 3, 16 (80.0%) of the decrease subgroup achieved PR, 4 (20.0%) SD. In the stable group, 21 (60.0%) demonstrated PR, 10 (28.6%) SD, and 4 (11.4%) PROG. The PSA increase group (*n* = 2) both exhibited PR responses. Effect size estimation using Cramér’s V indicated an increasing association between early PSA trend and PCWG3 response classification across treatment cycles. For Cycle 1, a small (statistically non-significant) association was observed (V = 0.17, p = 0.642). Cycle 2 showed a medium, statistically significant effect size (V = 0.41, *p* = 0.001). Cycle 3 data were too sparse for statistical testing due to limited patient numbers beyond the second cycle. Given the small sample sizes in particular for the increase group, the calculated Cramér’s V values should be interpreted with caution.

### Pearson correlation

Results of Pearson correlation between dPSA and rNadir are shown in Fig. 4. Sample sizes varied considerably across cycles and dPSA metrics. Figure 4 shows all ranging from minimum *N* = 3 (cycle 8, dPSA_1) to *N* = 76 (cycle 1, dPSA_1). For dPSA_1, statistically significant positive correlations with rNadir were observed in cycle 1 (r = 0.23, 95% CI: 0.00–0.43, *N* = 76, *p* = 0.047), cycle 2 (r = 0.44, 95% CI: 0.22–0.61, *N* = 66, p < 0.001), and cycle 3 (r = 0.27, 95% CI: 0.01–0.50, *N* = 57, *p* = 0.041). Correlations for cycle 1 and 2 remained statistically significant after FDR correction. For dPSA_2, the strongest correlations with rNadir were observed in the early treatment cycles. Cycle 1 demonstrated a moderate positive correlation (r = 0.54, 95% CI: 0.23–0.75, *N* = 31, *p* = 0.002), while cycle 2 showed a strong positive correlation (r = 0.82, 95% CI: 0.64–0.92, *N* = 25, p < 0.001). Both correlations retained statistical significance after FDR correction. Cycle 4 showed a moderate positive correlation to dPSA_2 (r = 0.38, 95% CI: − 0.05–0.69, *N* = 22, *p* = 0.084), though this did not reach statistical significance after correction. For dPSA_3, significant positive correlations with rNadir were found in cycles 1 (r = 0.52, 95% CI: 0.10–0.78, *N* = 20, *p* = 0.018) and cycle 2 (r = 0.46, 95% CI: 0.03–0.74, *N* = 21, *p* = 0.037), both statistically significant also after FDR correction. Cycle 4 showed a moderate correlation (r = 0.48, 95% CI: − 0.04–0.80, *N* = 15, *p* = 0.068) that approached but did not reach statistical significance. No statistically significant correlations were observed in cycles 5 through 8 for any dPSA metric after FDR correction, though sample sizes were substantially reduced in these later cycles (*N* ≤ 37).

### Kaplan–Meier survival analysis

Figure 5 shows the Kaplan–Meier survival analysis. It assesses the association between PSA kinetics during the first treatment cycle and OS. Patients were stratified according to their PSA trend classification (Decrease, Stable, or Increase) for each of the three dPSA metrics in cycle 1 (dPSA_1, dPSA2, dPSA3). For the dPSA_1 trend, median OS differed considerably across groups: Decrease 399 days (95% CI: 268–529), Stable 405 days (95% CI: 323–534), and Increase 225 days (95% CI: 58–308). Pairwise log-rank testing revealed no statistically significant difference between Decrease and Stable groups (*p* = 0.655), while the Increase group demonstrated significantly shorter OS compared to both Decrease (*p* = 0.019) and Stable groups (*p* = 0.003). dPSA_2 trend in cycle 1 showed a median OS of 406 days (95% CI: 324–447) for Decrease, 341 days (95% CI: 225–422) for Stable, and 206 days (95% CI: 113–394) for Increase. The Increase group showed a statistically significant shorter survival compared to the Decrease group (*p* = 0.045). No significant differences were observed between Decrease versus Stable, or Stable versus Increase. dPSA_3 trend in cycle 1 lead to a median OS of 380 days (95% CI: 165–460) for Decrease, 341 days (95% CI: 158–422) for Stable, and 173 days (95% CI: 173–347) for Increase. No statistically significant differences in OS were detected between any trend groups. The limited sample size however, particularly in the Increase group (*N* = 2), reduced statistical power for this analysis.

### Multivariable cox regression analysis

Multivariable Cox proportional hazards regression was performed to evaluate the independent prognostic value of PSA kinetics during cycle 1 while adjusting for established clinical covariates (Gleason score, baseline PSA, and age at diagnosis). Separate models were fitted for each dPSA metric in cycle 1 (Fig. 6). Of the 76 patients in the total cohort, complete baseline covariate data were available for 67 patients. Missing data comprised 7 patients lacking Gleason score, 1 patient lacking age at diagnosis, and 1 patient missing both covariates. Sample sizes for individual Cox models varied depending on the availability of the respective dPSA: *N* = 67 for dPSA_1, *N* = 28 for dPSA_2, and *N* = 18 for dPSA_3.

The dPSA_1 metric showed a HR of 7.89 (95% CI: 0.62–100.00, *p* = 0.111), suggesting a trend toward increased mortality risk with higher dPSA_1 values, though this did not reach statistical significance. Among the clinical covariates, Gleason score demonstrated a significant association with mortality (HR = 1.34, 95% CI: 1.03–1.74, *p* = 0.029), indicating that each one-point increase in Gleason score was associated with a 34% increase in hazard. Baseline PSA (HR = 1.00, 95% CI: 1.00–1.00, *p* = 0.050) and age at diagnosis (HR = 1.01, 95% CI: 0.98–1.04, *p* = 0.578) both had hazard ratios close to 1, indicating minimal independent association with mortality. The dPSA_2 model (*N* = 28) emerged as a statistically significant independent predictor of mortality (HR = 4.72, 95% CI: 1.35–16.44, *p* = 0.015). None of the clinical covariates reached statistical significance in this model: Gleason score (HR = 1.30, 95% CI: 0.79–2.12, *p* = 0.298), baseline PSA (HR = 1.00, 95% CI: 1.00–1.00, *p* = 0.840), and age at diagnosis (HR = 1.01, 95% CI: 0.96–1.06, *p* = 0.678). dPSA_3 model (*N* = 18) showed a HR of 3.81 (95% CI: 0.30–49.18, *p* = 0.305), with a wide confidence interval reflecting the limited sample size. This metric did not demonstrate a statistically significant association with mortality. Similarly, none of the clinical covariates achieved statistical significance under dPSA_3: Gleason score (HR = 1.45, 95% CI: 0.76–2.77, *p* = 0.261), baseline PSA (HR = 1.00, 95% CI: 1.00–1.00, *p* = 0.280), and age at diagnosis (HR = 1.01, 95% CI: 0.96–1.07, *p* = 0.696).

## Discussion

Since the official approval of PSMA therapy with [^177^Lu]Lu-PSMA-617 in 2022 for the treatment of mCRPC, there has been a steadily increasing number of treated patients worldwide. The complex procedure for [^177^Lu]Lu-PSMA-617 production, the relatively high treatment costs, and the often-limited capacity of nuclear medicine centers are important considerations in the accurate planning of PSMA-RLT. For example, in the US healthcare setting, treatment costs of [^177^Lu]Lu-PSMA-617 in mCRPC was double that of standard of care (USD 169,110 vs USD 85,398). The incremental cost effectiveness ratio was > USD 200,000 per quality adjusted life years. The maximum cost per cycle of ${}^{177}$ Lu-PSMA-617 to remain cost-effective is 38,826 USD [22].

Currently, around 10,000 to 15,000 doses of ${}^{177}$ Lu at 7.4 GBq are administered annually and there is already a significant shortage. ${}^{177}$ Lu production requires highly specialized high-flux neutron facilities.

Current nuclear reactor facilities cannot be relied upon for uninterrupted supply while construction of new ones demands at least 10 years [22]. Therefore, the nuclear medicine team strives to avoid cancellations of a scheduled PSMA-RLT due to any sudden contraindications, in part due to high cost of an unused [^177^Lu]Lu-PSMA-617 production. As such, early prediction of failure to respond [^177^Lu]Lu-PSMA-617 RLT would reduce unnecessary costs and help streamline department’s planning.

One of the most important parameters for monitoring response to PSMA-RLT is the progression of PSA levels. Earliest possible detection of relevant biochemical progression would provide valuable information which may drive appropriate decisions during follow-up. To date, several studies examining the potential of PSA changes measured across multiple [^177^Lu]Lu-PSMA-ligand cycles for predicting overall survival (OS) and progression-free survival (PFS) have concurred in showing that any PSA decline is promising for better outcome, whereas any PSA increase of ≥ 25% from pretreatment baseline levels is a significant predictor of poor response to therapy and shorter OS [8, 10, 23–26]. A previous study showed a positive linear relationship between higher rates of PSA decline after 1–2 cycles and OS, but the optimal threshold for defining a prompt PSA decline remains unclear [11].

To our best knowledge, there are no previous studies investigating the prompt PSA changes within a few days from the first RLT cycle as a predictor for biochemical response in consecutive therapy cycles, the Nadir (best PSA response), and OS.

Our study revealed significant correlations between prompt PSA changes on the second day (dPSA_1 Eq. 1) after injection of [^177^Lu]Lu-PSMA-ligands at cycles 1 and 2, with the individual Nadir response (Fig. 4). The main novelty of our results lies in our consideration of the predictive value of prompt PSA changes extending from the first to the third RLT cycle. Our findings of correlations between early PSA changes and subsequent Nadir outcomes suggest that dynamics of the early PSA response may be a useful indicator for predicting longer-term treatment outcomes. Measuring PSA levels two days after injection of the [^177^Lu]Lu-PSMA-ligand can be easily implemented in an inpatient setting and is therefore feasible in countries where the therapy must be administered on an inpatient basis according to the national authorities. On the other hand, for patients in countries where the therapy is approved for outpatient use, having their PSA level measured may cause discomfort for the patients.

In Kaplan–Meier analysis (Fig. 5), dPSA_1 significantly differentiated survival between patients with increasing versus stable or decreasing PSA trends, highlighting its potential early prognostic value. Although this association did not remain significant in the multivariable Cox model (Fig. 6), dPSA_1 showed the highest HR among the tested metrics. dPSA_2 and dPSA_3 showed weaker and less consistent associations with overall survival.

The predictive strength of dPSA_1 for PCWG3 classification is limited (Fig. 3). It remains to be seen whether larger datasets of patients can confirm the observed effect.

Our study has several limitations that should be acknowledged. First, its retrospective, single-center design may introduce selection bias and limit the generalizability of the findings. The sample size, though comparable to similar PSMA-RLT studies, was relatively small, especially in later treatment cycles, reducing statistical power for subgroup analyses and survival modeling. Additionally, all patients were hospitalized according to national regulations, which may not reflect outpatient treatment settings in other countries where PSMA-RLT is administered differently. The reliance on PSA kinetics as a surrogate marker of treatment response, without consistent integration of imaging or clinical endpoints such as radiographic progression-free survival, represents another limitation. Finally, while the early post-treatment PSA measurements offer a promising and practical prognostic tool, their biological basis remains uncertain and should be confirmed in larger, prospective multicenter trials with standardized protocols.

Further, there are known exceptions to our dPSA approach—namely patients who, despite rising PSA levels after the first cycle, still respond to the therapy after a delayed period [26, 27]. Nevertheless, the information provided by an early PSA measurement can help to more closely monitor potential non-responders and thereby reduce the risk of mismanagement or treatment planning failure.

## Conclusion

Patients with decreased or stable PSA levels according to a stringent threshold of 10% two days after the first cycle of PSMA-RLT therapy not only showed higher rates of favorable responses according to biochemical PCWG3 criteria, but also had prolonged overall survival compared to those with promptly increasing PSA levels. Additionally, the early PSA decline correlated with $PS{A}_{nadir}$ during the first three therapy cycles. These findings suggest that early PSA kinetics serve as a potentially valuable prognostic marker, enabling clinicians to predict treatment response. Patients with PSA increase two days after the first cycle of PSMA-RLT are at a significantly higher risk of being non-responders to treatment. They require closer monitoring and follow-up in order to reduce the risk of late cancellations of a scheduled PSMA-RLT due to any sudden contraindications thereby reducing unnecessary costs and helping streamline department’s planning.

## Supplementary Information

Below is the link to the electronic supplementary material.

Supplementary file1 (DOCX 36 KB)

## Data Availability

The underlying data of this evaluation can be requested from the corresponding author.
