# Supplementary material for: Prompt PSA changes as a prognostic marker for response to PSMA-radioligand therapy
Source: Eur J Nucl Med Mol Imaging. 2026 Jan 23;53(6):3518–28. doi: 10.1007/s00259-026-07772-y (PMC13649826; doi:10.1007/s00259-026-07772-y)
Supplement: Supplementary file 1 [file 259_2026_7772_MOESM1_ESM.docx]

# Appendix

Suppl-Table 1: PSA changes, split by cycle, and dPSA trend (decrease, stable, increase). dPSA: Delta PSA between two time points, we refer to equation 1, 2 and 3 in the main manuscript.

| **Cycle** | **dPSA** | **n per dPSA** | **Decrease** | **Stable** | **Increase** |
| --- | --- | --- | --- | --- | --- |
| 1 | 1 | 76 | n=32 median=-17.4% 95%CI=[-33.8%,-10.4%] | n=37 median=-3.8% 95%CI=[-9.8%,7.1%] | n=7 median=18.4% 95%CI=[11.6%,25.4%] |
| 1 | 2 | 31 | n=12 median=-19.9% 95%CI=[-59.7%,-10.9%] | n=7 median=-2.3% 95%CI=[-9.1%,4.8%] | n=12 median=31.4% 95%CI=[16.1%,131.4%] |
| 1 | 3 | 20 | n=10 median=-34.3% 95%CI=[-53.6%,-11.4%] | n=8 median=-1.8% 95%CI=[-9.1%,7.9%] | n=2 median=35.1% 95%CI=[13.4%,56.8%] |
| 2 | 1 | 66 | n=29 median=-15.2% 95%CI=[-28.9%,-10.1%] | n=36 median=-4.0% 95%CI=[-9.8%,6.5%] | n=1 13.3% |
| 2 | 2 | 25 | n=12 median=-25.2% 95%CI=[-44.5%,-12.5%] | n=8 median=3.2% 95%CI=[-7.7%,9.1%] | n=5 median=32.3% 95%CI=[14.6%,43.3%] |
| 2 | 3 | 21 | n=9 median=-30.1% 95%CI=[-48.4%,-17.7%] | n=7 median=3.9% 95%CI=[-7.3%,9.0%] | n=5 median=49.8% 95%CI=[28.7%,77.6%] |
| 3 | 1 | 57 | n=20 median=-16.4% 95%CI=[-40.5%,-10.6%] | n=35 median=-4.6% 95%CI=[-9.2%,7.9%] | n=2 median=25.4% 95%CI=[16.2%,34.5%] |
| 3 | 2 | 19 | n=8 median=-21.6% 95%CI=[-39.0%,-12.3%] | n=4 median=1.5% 95%CI=[-6.3%,2.9%] | n=7 median=35.0% 95%CI=[14.2%,70.8%] |
| 3 | 3 | 13 | n=4 median=-27.2% 95%CI=[-34.9%,-20.2%] | n=5 median=2.2% 95%CI=[-6.3%,5.3%] | n=4 median=58.8% 95%CI=[14.1%,128.7%] |
| 4 | 1 | 47 | n=17 median=-15.5% 95%CI=[-27.9%,-10.4%] | n=28 median=-3.8% 95%CI=[-10.0%,6.5%] | n=2 median=21.5% 95%CI=[13.3%,29.7%] |
| 4 | 2 | 22 | n=11 median=-21.9% 95%CI=[-41.9%,-12.3%] | n=6 median=-7.1% 95%CI=[-9.4%,4.9%] | n=5 median=20.8% 95%CI=[13.4%,48.3%] |
| 4 | 3 | 15 | n=5 median=-15.2% 95%CI=[-43.1%,-10.5%] | n=6 median=2.7% 95%CI=[-3.6%,6.3%] | n=4 median=28.2% 95%CI=[16.4%,40.7%] |
| 5 | 1 | 37 | n=19 median=-14.6% 95%CI=[-24.0%,-11.1%] | n=17 median=-3.8% 95%CI=[-9.4%,8.7%] | n=1 17.9% |
| 5 | 2 | 13 | n=3 median=-18.1% 95%CI=[-21.3%,-17.9%] | n=3 median=1.8% 95%CI=[-6.3%,2.0%] | n=7 median=26.9% 95%CI=[14.0%,72.3%] |
| 5 | 3 | 10 | n=4 median=-35.2% 95%CI=[-59.8%,-11.6%] | n=5 median=2.2% 95%CI=[-5.2%,5.3%] | n=1 39.7% |
| 6 | 1 | 34 | n=11 median=-20.4% 95%CI=[-36.4%,-12.4%] | n=20 median=-4.1% 95%CI=[-9.0%,8.7%] | n=3 median=19.8% 95%CI=[11.6%,61.9%] |
| 6 | 2 | 14 | n=5 median=-15.2% 95%CI=[-54.5%,-12.8%] | n=3 median=5.1% 95%CI=[4.0%,5.5%] | n=6 median=28.7% 95%CI=[13.0%,60.4%] |
| 6 | 3 | 13 | n=2 median=-14.9% 95%CI=[-18.5%,-11.3%] | n=4 median=-3.8% 95%CI=[-9.1%,5.9%] | n=7 median=44.6% 95%CI=[19.3%,121.9%] |
| 7 | 1 | 6 | n=1 -23.1% | n=1 7.2% | n=4 median=20.3% 95%CI=[19.5%,54.4%] |
| 7 | 2 | 1 | n=0 | n=0 | n=1 25.7% |
| 7 | 3 | 1 | n=1 -16.9% | n=0 | n=0 |
| 8 | 1 | 3 | n=1 -12.5% | n=0 | n=2 median=37.7% 95%CI=[19.3%,56.2%] |
| 8 | 2 | 2 | n=0 | n=1 2.9% | n=1 83.6% |
| 8 | 3 | 2 | n=1 -37.2% | n=1 -2.8% | n=0 |

Suppl-Table 2: Patients who discontinued therapy, split by dPSA trend (decrease, stable, increase). dPSA: Delta PSA between two time points, we refer to equation 1, 2 and 3 in the main manuscript.

| **Cycle** | **dPSA** | **n per dPSA** | **Decrease** | **Stable** | **Increase** |
| --- | --- | --- | --- | --- | --- |
| 1 | 1 | 10 | n=4 median=-13.7% 95%CI=[-23.8%,-10.3%] | n=5 median=-5.4% 95%CI=[-6.4%,4.1%] | n=1 18.4% |
| 1 | 2 | 3 | n=0 | n=0 | n=3 median=41.4% 95%CI=[27.6%,141.5%] |
| 1 | 3 | 1 | n=0 | n=1 8.6% | n=0 |
| 2 | 1 | 10 | n=3 median=-15.1% 95%CI=[-17.2%,-10.3%] | n=7 median=-2.2% 95%CI=[-7.3%,3.9%] | n=0 |
| 2 | 2 | 2 | n=0 | n=0 | n=2 median=32.5% 95%CI=[25.4%,39.6%] |
| 2 | 3 | 1 | n=0 | n=0 | n=1 49.8% |
| 3 | 1 | 9 | n=2 median=-16.0% 95%CI=[-19.1%,-12.9%] | n=7 median=-1.3% 95%CI=[-8.8%,6.9%] | n=0 |
| 3 | 2 | 4 | n=1 -25.2% | n=1 1.8% | n=2 median=23.2% 95%CI=[12.0%,34.5%] |
| 3 | 3 | 4 | n=1 -20.0% | n=2 median=3.8% 95%CI=[2.3%,5.4%] | n=1 14.0% |
| 4 | 1 | 10 | n=4 median=-13.1% 95%CI=[-16.5%,-10.3%] | n=6 median=-3.7% 95%CI=[-8.4%,2.3%] | n=0 |
| 4 | 2 | 5 | n=2 median=-25.1% 95%CI=[-30.5%,-19.8%] | n=1 -5.2% | n=2 median=20.3% 95%CI=[19.8%,20.8%] |
| 4 | 3 | 3 | n=0 | n=0 | n=3 median=32.8% 95%CI=[16.7%,41.0%] |
| 5 | 1 | 4 | n=2 median=-20.4% 95%CI=[-24.5%,-16.4%] | n=2 median=-6.1% 95%CI=[-7.7%,-4.5%] | n=0 |
| 5 | 2 | 2 | n=0 | n=0 | n=2 median=17.7% 95%CI=[13.4%,22.0%] |
| 5 | 3 | 2 | n=0 | n=2 median=-0.8% 95%CI=[-5.5%,3.8%] | n=0 |
| 6 | 1 | 26 | n=9 median=-20.4% 95%CI=[-37.3%,-12.5%] | n=14 median=-3.0% 95%CI=[-8.9%,8.8%] | n=3 median=19.8% 95%CI=[11.6%,61.9%] |
| 6 | 2 | 12 | n=3 median=-43.5% 95%CI=[-55.1%,-16.6%] | n=3 median=5.1% 95%CI=[4.0%,5.5%] | n=6 median=28.7% 95%CI=[13.0%,60.4%] |
| 6 | 3 | 11 | n=1 -11.1% | n=3 median=-4.4% 95%CI=[-9.2%,6.1%] | n=7 median=44.6% 95%CI=[19.3%,121.9%] |
| 7 | 1 | 3 | n=0 | n=1 7.2% | n=2 median=19.7% 95%CI=[19.5%,20.0%] |
| 7 | 2 | 0 | n=0 | n=0 | n=0 |
| 7 | 3 | 0 | n=0 | n=0 | n=0 |
| 8 | 1 | 2 | n=0 | n=0 | n=2 median=37.7% 95%CI=[19.3%,56.2%] |
| 8 | 2 | 1 | n=0 | n=0 | n=1 83.6% |
| 8 | 3 | 1 | n=1 -37.2% | n=0 | n=0 |
